# A Case of Late Recurrence of Colon Cancer After Curative Treatment

**DOI:** 10.7759/cureus.8083

**Published:** 2020-05-13

**Authors:** Alsadiq Al Hillan, Kadhim Al-Banaa, Mujtaba Mohamed, Fadi Hawa, Penny Turtel

**Affiliations:** 1 Internal Medicine, Jersey Shore University Medical Center, Neptune, USA; 2 Internal Medicine, Beaumont Hospital, Dearborn, USA; 3 Internal Medicine, St. Joseph Mercy Ann Arbor Hospital, Ann Arbor, USA; 4 Gastroenterology, Jersey Shore University Medical Center, Neptune, USA; 5 Gastroenterology, Monmouth Medical Center, Long Branch, USA

**Keywords:** colon cancer recurrence, colon cancer surveillance

## Abstract

Colorectal cancer (CRC) is the most common tumor type in both sexes combined in Western countries. Although screening programs, including the implementation of fecal occult blood test and colonoscopy, might reduce mortality by removing precursor lesions and making the diagnosis at an earlier stage. Unfortunately, ~25% to 40% will develop a tumor recurrence despite a curative operation. It is well-known that most recurrences occur within five years. There are a lot of solid guidelines for recurrence surveillance. We present a case of colon adenocarcinoma that underwent surgical resection of the descending colon with close recurrence surveillance follow-ups that showed normal carcinoembryonic antigen (CEA) for 12 years and then presented again with blood in stool and was found to have recurrent colon adenocarcinoma.

## Introduction

About 140,000 people in the United States are diagnosed annually with colorectal cancer [1]. Despite a potentially curative operation, nearly 10% of patients experience late recurrence of the disease five years after surgery [2]. The optimal strategy to accurately detect recurrences at the earliest stage remains controversial. The current recommendations for follow-up surveillance include a combination of history and physical examination, laboratory evaluation, imaging, and endoscopy on slightly varying schedules depending on the organization and stage of disease [3-11]. The early diagnosis of recurrence following surgical resection is a key determinant of survival. Up to 40% of patients with a locoregional disease will develop recurrent cancer, of which 90% will occur within five years [4-7]. There are no data for long-term recurrence. We present a case of recurrent colon adenocarcinoma after 12 years of remission.

## Case presentation

**Figure 1:**
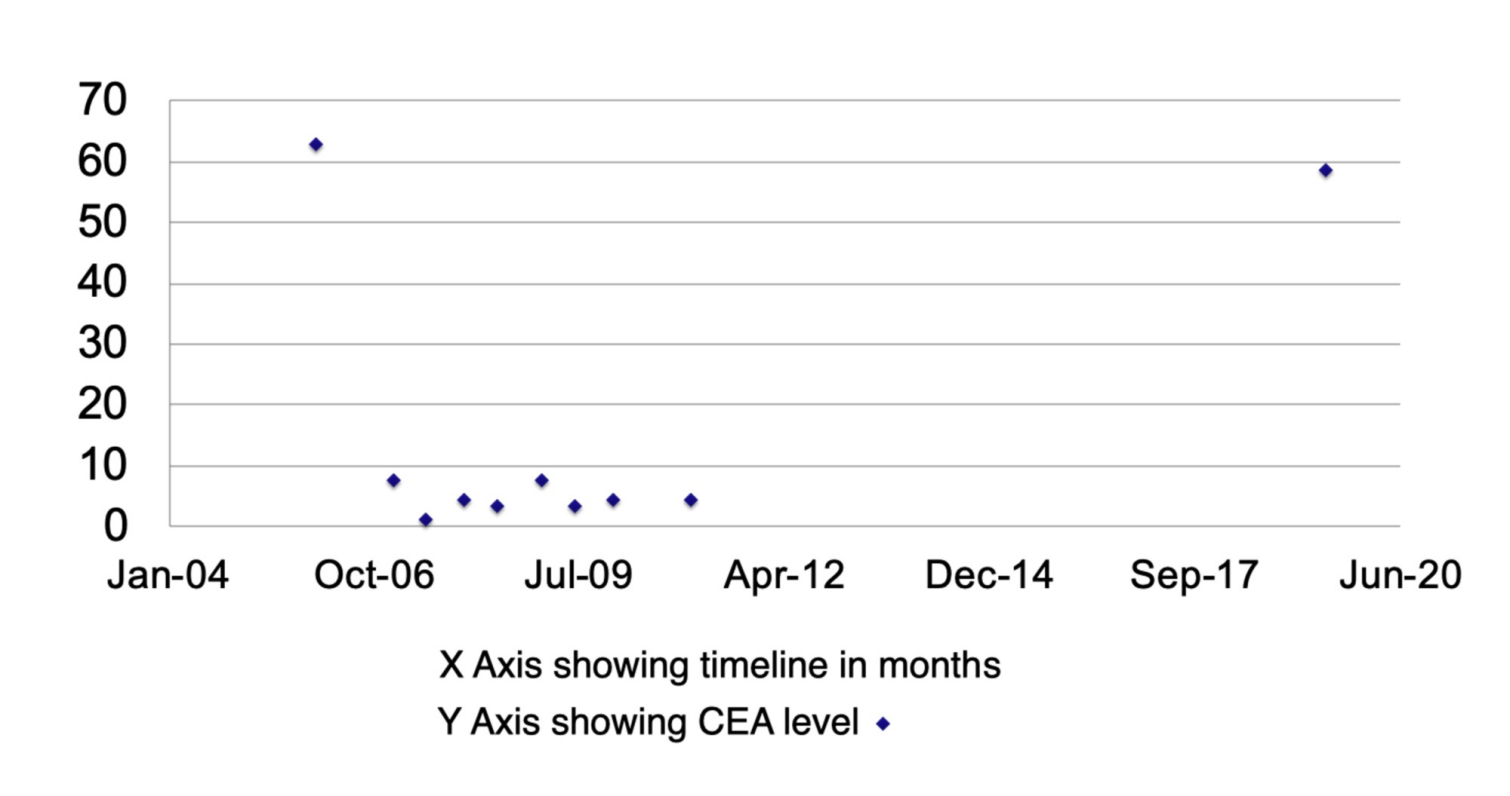
Patient CEA levels at the time of diagnosis and during follow-up CEA: carcinoembryonic antigen

A 70-year-old female with a past medical history of hypertension, hyperlipidemia, and moderately differentiated colon adenocarcinoma status post resection of the descending colon in 2006 and close follow-up with guideline-directed recurrence surveillance, including her last colonoscopy in February 2019 that was unremarkable, presented with complaints of weight loss and fatigue but no abdominal pain, nausea, vomiting, or change in bowel habits. Vital signs showed blood pressure 116/70 mmHg, respiratory rate 14 breath/minute, heart rate 74 beats/minute, and a temperature of 98.1 degrees Fahrenheit. The general physical examination was remarkable for pale conjunctivae. Abdominal examination revealed a soft abdomen with normal bowel sounds and no tenderness, guarding palpable masses, or organomegaly. Laboratory findings were remarkable for anemia, low ferritin, and elevated carcinoembryonic antigen (CEA) (Table 1, Figure 1).

**Table 1:** Laboratory findings

	Result	Reference Range
White blood cell count	9.1 K/uL	4.5 -11 K/uL
Hemoglobin	13.1 gm/dL	12 – 16 gm/dL
Hematocrit	43.1 %	35-48%
Platelet count	342 K/uL	140-450 K/uL
Blood urea nitrogen	14 mg/dL	5-25 mg/dL
Creatinine	0.9 md/dL	0.44-1 mg/dL
Erythrocyte sedimentation rate	14 mm/hr	15-20 mm/hr
C-reactive protein	0.62 mg/L	< 1 mg/L

**Figure 2:**
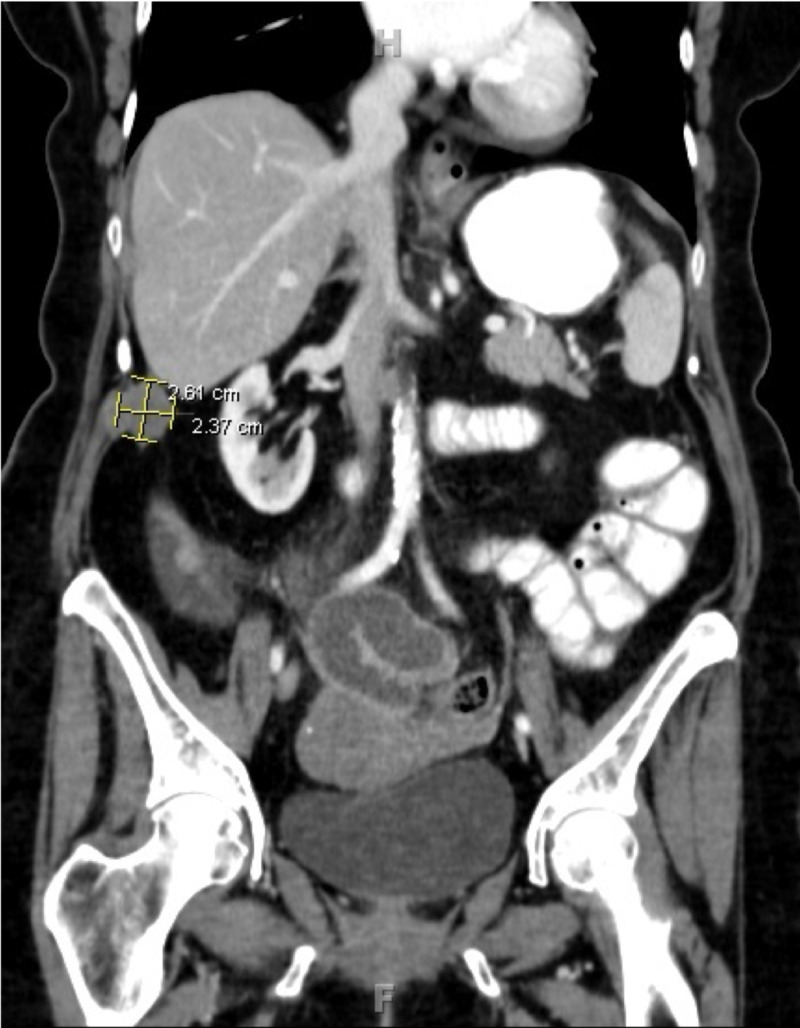
CT scan of the abdomen and pelvis with contrast showing right sub-hepatic omental mass measuring 2.63 X 2.37 cm CT: computed tomography

As colonoscopy failed to detect colon cancer recurrence in our patient, a computed tomography (CT) scan of the chest, abdomen, and pelvis revealed omental masses with the largest measuring 2.36 x 2.61 cm (Figure 2). CT-guided core biopsy of the omental mass showed metastatic colonic adenocarcinoma that had 60% gland formation (moderately differentiated) with no desmoplasia or desmoplastic reaction. Tumor cells were immunoreactive for cytokeratins 20 and CDX2 (homeobox protein responsible for the maintenance of the intestinal phenotype) while negative for cytokeratins 7, estrogen receptor, and paired‑box 8 (markers for ovarian cancer). The morphological and histochemical pattern mentioned above strongly supports the locoregional recurrence of colonic primary adenocarcinoma rather than primary ovarian cancer. The patient was started on chemotherapy for several months now.

## Discussion

Surgical resection is the mainstay of colorectal cancer treatment [12]. Depending on the Tumour, Node, Metastasis (TNM) stage at diagnosis, adjuvant therapy may be added in addition to the surgery [13]. The current recommendations for follow-up surveillance include a combination of history and physical examination, and laboratory evaluation (including measurement of CEA, imaging, and endoscopy on slightly varying schedules depending on the organization and stage of disease) [3-11].

The American Cancer Society, the US Multi-Society Task Force on Colorectal Cancer, and the American Society of Colon and Rectal Surgeons recommend that patients who have undergone curative resection of either colon or rectal cancer receive their first surveillance colonoscopy one year after surgery (or one year after the clearing perioperative colonoscopy). After the one-year colonoscopy, the interval to the next colonoscopy should be three years (i.e., four years after surgery or perioperative colonoscopy) and then five years. Subsequent colonoscopies should occur at five-year intervals [13-15]. Surveillance colonoscopy should be discontinued in patients with advanced age or commodities that set their life expectancy to less than 10 years according to the clinician's judgment [15]. Office visits should be done on a regular basis and include CEA testing. For patients with stage II or III colorectal cancers, the frequency should be every three to six months for the first two years and then twice a year for a total of five years [13-14]. They also strongly recommend, as part of the surveillance, radiographic imaging with cross-sectional chest and abdominopelvic imaging (e.g., CT or magnetic resonance imaging (MRI) scans) to be done annually for five years based on moderate-quality evidence from randomized clinical trials [13-14]. As repeated surveillance colonoscopy failed to detect colon cancer recurrence in our patient, and given that both CEA measurement and imaging studies were indicative of colon cancer recurrence at the time of diagnosis, we would argue that continuing CEA measurement and imaging studies for longer durations than what guidelines suggested, they can detect recurrence when colonoscopy fails. Factors to consider when recommending surveillance include patient comorbidity, activity level, age, patient preference, and compliance. Also, the overall success of surveillance for the early detection of curable recurrence will depend on a commitment for both providers and patients to adhere to the surveillance schedule. Our patient had a very close follow-up with her gastroenterologist and the oncologist but even after undergoing guideline-recommended surveillance, this raises the question of what would be the next guidelines if we start seeing more of these late recurrence cases. Our extensive literature search didn't reveal similar cases that had recurrence after 10 years, so we believe that our case report will be a valuable addition for future studies that might focus on looking at extending the surveillance in regards to costs/benefits, risk of radiation, and the hassle of prolonged follow-up.

## Conclusions

CEA measurement and imaging studies were able to guide the diagnosis of colon cancer recurrence after 12 years of remission when colonoscopy didn't indicate that. Some patients might benefit from prolonged surveillance by extending CEA measurement and imaging studies. Further studies would be needed to identify those who might be considered a higher risk for late recurrence.
